# Glycemic Burden and Clinical Outcomes of Early Stage Hepatocellular Carcinoma after Curative Treatment

**DOI:** 10.3390/cancers16152652

**Published:** 2024-07-26

**Authors:** Hyun Joo Lee, Moon Seok Choi, Byeong Geun Song, Won Seok Kang, Geum Youn Gwak, Myung Ji Goh, Yong Han Paik, Joon Hyeok Lee, Dong Hyun Sinn

**Affiliations:** Division of Gastroenterology and Hepatology, Department of Internal Medicine, Samsung Medical Center, Sungkyunkwan University School of Medicine, Seoul 06351, Republic of Korea; hyunjoo1025.lee@samsung.com (H.J.L.);

**Keywords:** hepatocellular carcinoma, glycemic burden, diabetes mellitus, HbA1c, IGF-1

## Abstract

**Simple Summary:**

Early-stage hepatocellular carcinoma (HCC) is notorious for its high recurrence rate even after curative treatment. Several studies have suggested the association between diabetes mellitus (DM) and the risk of HCC. However, current evidence regarding the impact of glycemic burden on the outcomes of HCC is still limited. The present study provides an important insight into the relationship between glycemic burden and outcomes of early-stage HCC. Lower glycemic burden was an independent factor associated with better overall survival as well as lower recurrence in early-stage HCC. Moreover, there was a dose–response relationship between recurrence/overall survival and glycemic burden. Good glycemic control should be considered as a significant part of HCC management.

**Abstract:**

Early-stage hepatocellular carcinoma (HCC) is still difficult to cure for its high recurrence rate. This study aimed to examine whether glycemic burden management could be one way to improve outcomes of early-stage HCC. A total of 137 very early or early-stage HCC patients who underwent resection or ablation at Samsung Medical Center and had glycemic burden assessment were analyzed. Glycemic burden was assessed using hemoglobin A1c (HbA1c) level. Outcomes were recurrence and overall survival. Risks of recurrence and overall survival were compared according to glycemic burden using a cut-off point of 6.5% or two cut-off points of 6.0% and 7.5%. Overall, 51 (37.2%) patients experienced HCC recurrence. The adjusted hazard ratio (aHR) for recurrence comparing patients with HbA1c > 6.5% to those with HbA1c ≤ 6.5% was 2.66 (95% CI: 1.26–5.78). The risk of recurrence increased in a dose-dependent manner by glycemic burden; aHR for 6.0 < HbA1c ≤ 7.5%: 2.00 (95% CI: 0.78–5.55); aHR for HbA1c > 7.5%: 6.05 (95% CI: 2.31–17.5). Mortality was observed in 16 (11.7%) patients. The risk of mortality was higher for HbA1c > 6.5% than for HbA1c ≤ 6.5% (aHR: 2.33; 95% CI: 1.10–5.08). There was also a dose–response relationship between overall survival and glycemic burden. Glycemic burden assessed using HbA1c level was significantly associated with outcomes of early-stage HCC patients. Good glycemic control could be a therapeutic goal to improve clinical outcomes in these populations.

## 1. Introduction

Hepatocellular carcinoma (HCC) is one of the most common malignancies and the leading cause of cancer death worldwide [1]. For early-stage HCC, liver resection, liver transplantation, and local ablation therapy such as radiofrequency ablation (RFA) are considered the first-line of curative treatment [2,3]. However, early-stage HCC is notorious for its high recurrence rate even after curative treatment [4]. The recurrence rate of HCC at 5 years after curative treatment is around 50–60%, which considerably restricts long-term survival of early-stage HCC patients [5,6,7]. Thus, effective adjuvant therapy is urgently required, but there is still no widely validated adjuvant therapy [4]. Although adjuvant immunotherapy with cytokine-induced killer cells has shown promising results in a prospective randomized controlled trial [8], its high cost remains a problem. Randomized controlled phase 3 studies on adjuvant therapies using immune checkpoint inhibitors are underway, with their results eagerly awaited [9]. However, high costs and possible side effects associated with immune checkpoint inhibitors are expected to remain problems. Herein, identifying potentially modifiable risk factors for HCC recurrence is still an unmet clinical need.

Diabetes mellitus (DM), a common metabolic disorder, plays an important role in HCC development. Several previous studies have suggested an increased risk of HCC in patients with DM [10,11,12,13]. By meta-analyses of cohort studies, the mortality as well as the incidence of HCC were higher in patients with DM than patients without DM [14,15,16]. DM was also reported to be an established risk factor for the rapid progression of non-alcoholic fatty liver disease (NAFLD) to cirrhosis, or HCC [11,17,18]. On the other hand, antidiabetic treatment with metformin was significantly associated with a reduced risk of HCC and not only prolonged overall survival (OS), but also decreased the recurrence rate for HCC patients with DM after curative therapy [19,20].

These findings suggest that DM can be a modifiable risk factor to improve the outcome of early-stage HCC. However, current evidence regarding the clinical significance of DM on the outcome of early-stage HCC patients shows conflicting results. While some studies have indicated that DM is associated with a poor outcome, other studies have found no association between DM and clinical outcomes in early-stage HCC patients [21,22,23]. In fact, the effects of glycemic burden on the recurrence and survival of early-stage HCC patients after curative treatment have not been well studied yet. Therefore, this study aimed to investigate whether glycemic burden could affect the long-term outcomes of HCC patients after curative treatment such as resection or ablation.

## 2. Materials and Methods

### 2.1. Study Design, Participants, and Variables

**Figure 1:**
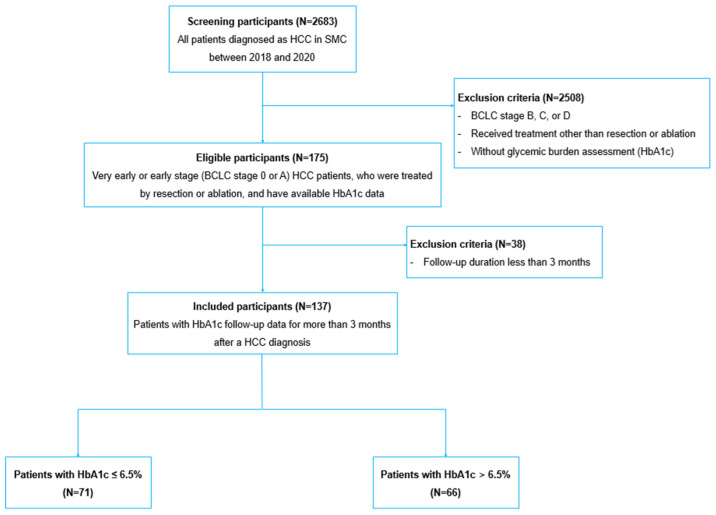
Flow chart of the study design. HCC = hepatocellular carcinoma; SMC = Samsung Medical Center; BCLC = Barcelona clinic liver cancer; HbA1c = hemoglobin A1c.

In this retrospective cohort study, a total of 2683 consecutive, newly diagnosed HCC patients at Samsung Medical Center, Seoul, South Korea, between 2018 and 2020 were screened. Of them, 175 patients with very early or early-stage (BCLC stage 0 or A) HCC who were treated by resection or ablation and had glycemic burden assessment were included. After excluding 38 patients with follow-up duration less than 3 months, 137 patients were finally analyzed (Figure 1). The present cohort study was approved by the institutional review board of Samsung Medical Center. No personal identification information was included in the data to protect the privacy of participants, and the requirement for informed consent was waived. All procedures involved in this study were performed in accordance with the relevant guidelines and regulations (Declaration of Helsinki).

The primary outcome was HCC recurrence during the follow-up period. The follow-up period was defined as the period from the date of diagnosis to the date of recurrence, last follow-up without HCC recurrence, or death, whichever came first. The secondary outcome was OS. The exposure was a glycemic burden. For confounders, we collected information on age, sex, body mass index (BMI), presence of hypertension and dyslipidemia, etiological liver disease, AST/ALT levels, platelet count, prothrombin time, total bilirubin levels, albumin levels, Child–Pugh score, tumor size, number of tumors, and AFP levels.

### 2.2. Glycemic Burden Assessment

Glycemic burden was determined using serum hemoglobin A1c (HbA1c) level. The index period was three months from the HCC diagnosis date. For patients who had multiple HbA1c measurements during the index period, the highest value was used. Patients were categorized into two groups according to HbA1c levels, i.e., the high glycemic burden (HbA1c > 6.5%) group and the low glycemic burden (HbA1c ≤ 6.5%) group. To see whether a dose–response relationship exists, we further subdivided patients into the following three groups according to glycemic burden: HbA1c ≤ 6.0% group, HbA1c = 6.0–7.5% group, and HbA1c > 7.5% group.

### 2.3. Statistical Analysis

Variables are described as mean ± standard deviation, median with interquartile range (IQR), frequencies, or percentages as appropriate. The difference between the two groups was compared using the Student’s *t*-test, Mann–Whitney U test, or Fisher’s test. The Kaplan–Meier method was utilized to plot survival curves or cumulative recurrence curves, and the log-rank test was used to compare differences in rates. The risk of HCC recurrence as well as OS according to glycemic burden was estimated by performing univariate and multivariate Cox regression analyses. In multivariate Cox regression analysis, age (per year), sex, tumor size (per cm), tumor number, Child–Pugh score (5 vs. 6–9), etiological liver disease (HBsAg positivity), and treatment method (resection vs. ablation) were adjusted. All reported *p*-values were two-sided, and the significance level was set at *p* < 0.05. Statistical analyses were performed with GraphPad Prism software v10.0.1 (GraphPad Software Inc., San Diego, CA, USA).

## 3. Results

### 3.1. Baseline Characteristics

In this study cohort, 71 patients had a low glycemic burden (HbA1c level ≤ 6.5%), whereas 66 patients had a high glycemic burden (HbA1c level > 6.5%). Baseline characteristics according to glycemic burden are shown in Table 1. Baseline characteristics except for etiological liver disease were similar between the two groups. The median age was 62 years (IQR 55.0–66.0) in the low glycemic burden group and 64 years (IQR 56.8–69.3) in the high glycemic burden group. The median follow-up duration was 3.7 years (IQR 3.1–4.6). The rate of surgical resection was higher than that of ablation in both groups. There was a statistically significant difference only in etiological liver disease. HBV was the most common etiology in both groups, and non-HBV and non-HCV causes were more common in the high glycemic burden group than the low glycemic burden group. Probably, NAFLD among non-HBV and non-HCV liver diseases may comprise a significant proportion of the etiologies of patients with higher HbA1c levels.

### 3.2. Impact of Glycemic Burden on HCC Recurrence

**Figure 2:**
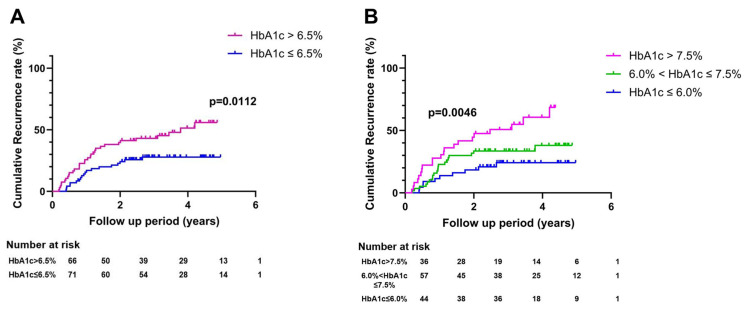

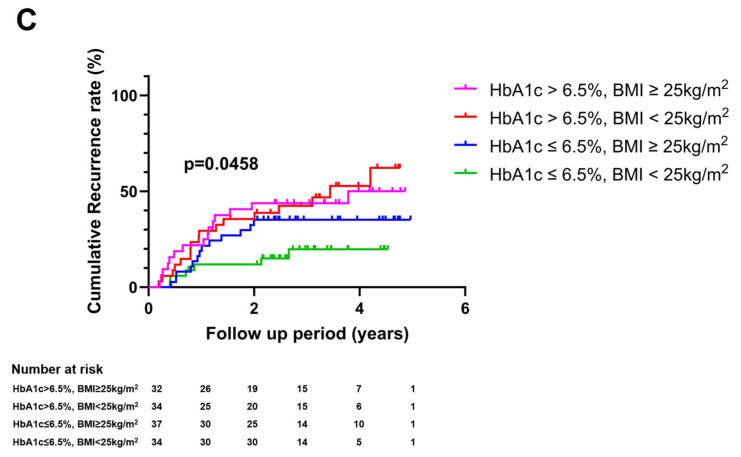
Cumulative recurrence rate curves by glycemic control status. (**A**) Recurrence rate curves were compared between two groups categorized using a cut-off point of 6.5%. (**B**) Recurrence rate curves were compared among three groups categorized using two cut-off points of 6.0% and 7.5%. (**C**) Recurrence rate curves were shown comparing four groups subdivided by glycemic burden and body mass index (BMI).

During the follow-up period, 51 (37.2%) patients experienced HCC recurrence. HCC recurrence rate was higher in patients with a high glycemic burden than in those with a low glycemic burden (55.8% vs. 27.8% at 5 years, *p* = 0.0112, Figure 2A). Based on unadjusted Cox regression analysis, the risk of HCC recurrence was higher for patients with a high glycemic burden than for patients with a low glycemic burden (Table 2). The adjusted hazard ratio (aHR) for recurrence comparing patients with a high glycemic burden to those with a low glycemic burden was 2.66 (95% CI: 1.26–5.78). When rates of HCC recurrence were compared according to three categories (HbA1c ≤ 6.0%, HbA1c = 6.0–7.5%, and HbA1c > 7.5%), the recurrence rate was the highest in patients with the highest glycemic burden (HbA1c > 7.5%) and the lowest in patients with the lowest glycemic burden (HbA1c ≤ 6.0%) (Figure 2B, *p* = 0.0046). The risk of recurrence by glycemic burden increased in a dose-dependent manner (Table 2). We additionally compared the recurrence rates after subdividing the subjects based on BMI. When glycemic burden was high (HbA1c > 6.5%), the recurrence rate was higher in patients with BMI < 25kg/m^2^ than patients with BMI ≥ 25kg/m^2^ (Figure 2C, *p* = 0.0458). In contrast, when glycemic burden was low (HbA1c ≤ 6.5%), the recurrence rate was lower in patients with BMI < 25kg/m^2^ than in patients with BMI ≥ 25kg/m^2^.

### 3.3. Impact of Glycemic Burden on HCC Mortality and Recurrence-Free Survival

**Figure 3:**
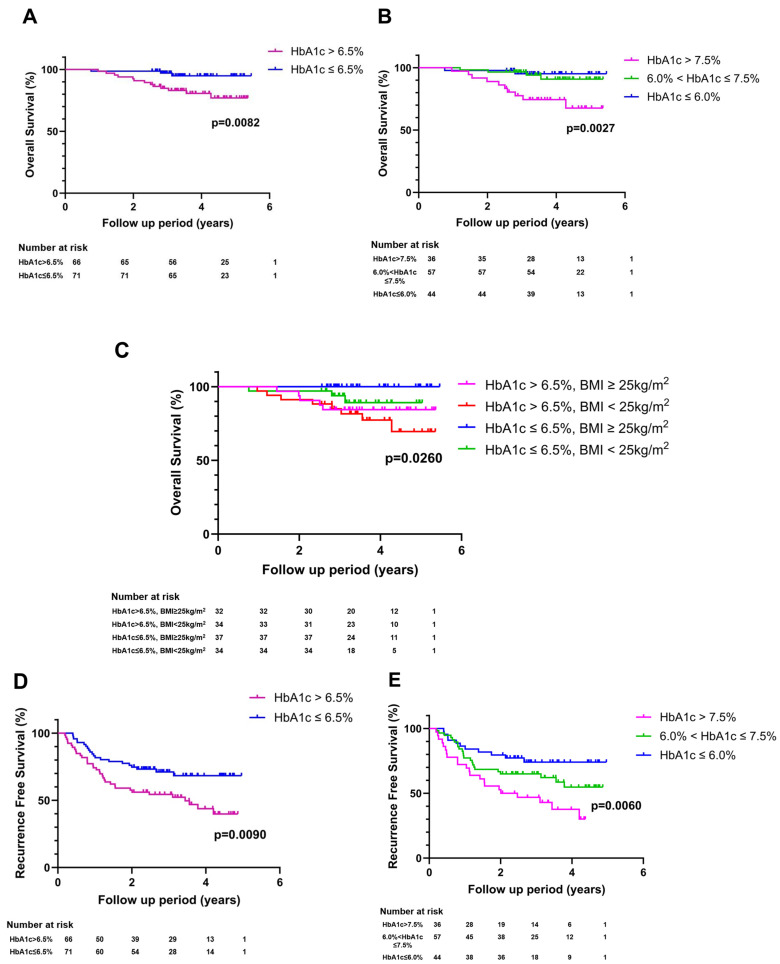
Overall survival curves and recurrence-free survival curves by glycemic control status. (**A**) Overall survival curves were compared between two groups categorized using a cut-off point of 6.5%. (**B**) Overall survival curves were compared among three groups categorized using two cut-off points of 6.0% and 7.5%. (**C**) Overall survival curves were compared among four groups subdivided by glycemic burden and BMI. (**D**) Recurrence-free survival curves were shown comparing two groups divided according to a cut-off point of 6.5%. (**E**) Recurrence-free survival curves were shown comparing three groups subdivided using two cut-off points of 6.0% and 7.5%.

Mortality was observed in 16 (11.7%) patients. OS was better for patients with HbA1c ≤ 6.5% than for patients with HbA1c > 6.5% (94.8% vs. 76.9% at 5 years, *p* = 0.0082, Figure 3A). The risk of mortality was higher for patients with a high glycemic burden (HbA1c > 6.5%) than for those with a low glycemic burden (HbA1c ≤ 6.5%) (aHR: 2.33; 95% CI: 1.10–5.08, Table 3). When glycemic burden was sorted into three groups, patients with the highest category of glycemic burden (HbA1c > 7.5%) showed the highest risk of mortality, while patients with the lowest category of glycemic burden (HbA1c ≤ 6.0%) showed the lowest risk of mortality (Figure 3B and Table 3). We further made subgroups based on BMI and repeated the analysis. Patients with BMI < 25kg/m^2^ showed worse OS than patients with BMI ≥ 25kg/m^2^, whether glycemic burden was high or low (Figure 3C, *p* = 0.0260). Likewise with OS, recurrence-free survival (RFS) was also better for patients with HbA1c ≤ 6.5% than for patients with HbA1c > 6.5% (68.3% vs. 39.7% at 5 years, *p* = 0.0090, Figure 3D). As glycemic burden was categorized into three groups, patients with the highest category of glycemic burden (HbA1c > 7.5%) showed the worst RFS, whereas patients with the lowest category of glycemic burden (HbA1c ≤ 6.0%) showed the best RFS (Figure 3E, *p* = 0.0060).

## 4. Discussion

Resection and ablation are the first-line treatment recommendations for very early and early-stage HCC, and the survival of very early or early-stage HCC is generally good. But, even after curative treatment with resection or ablation for early-stage HCC, the recurrence rate is still very high [24]. In a study of early-stage HCC in our center, the recurrence rate was 60.3% at 5 years and 71% at 10 years [5]. Hence, efforts are required to decrease HCC recurrence risk. In this perspective, identifying modifiable risk factors to improve treatment outcomes is needed.

This study revealed that glycemic burden assessed using HbA1c levels is an independent factor associated with tumor recurrence in patients with early-stage HCC. The association between glycemic burden and tumor recurrence remained significant even after adjusting for potential mediators or confounding factors. Moreover, the observed association tended to be dose-dependent. Similarly, the OS was also poorer for patients with a high glycemic burden, and patients with a higher glycemic burden had a higher risk of mortality than patients with a lower glycemic burden.

Although the exact mechanism by which a higher glycemic burden is associated with poorer outcomes in HCC patients has not been unveiled, there are several pieces of evidence that suggest a causal relationship between glycemic burden and poorer clinical outcome. Various cohort studies and meta-analyses have shown that DM is a risk factor for increased incidence and recurrence as well as poor prognosis in different types of cancers such as pancreatic, colorectal, gastric, and ovarian cancers [25,26,27,28,29,30,31]. DM is also known to contribute to hepatocarcinogenesis, and IGF-1 plays a pivotal role in hyperglycemia-induced hepatocarcinogenesis [32]. Insulin resistance and hyperglycemia stimulate IGF-1 signaling, and IGF-1 then activates oncogenic signaling pathways such as PI3K/Akt/mTOR, JNK/MAPK, Wnt, and Ras/MEK/ERK, which promote cell proliferation and angiogenesis and inhibit apoptosis, eventually fostering the development of HCC [33]. In addition, accumulated reactive oxygen species (ROS) act as major mediators for the activation of pro-inflammatory signaling such as MAPK, JAK/STAT, or NF-κB signaling, and pro-inflammatory cytokines like TNF-α, IL-6, or IL-1β and DNA damage are responsible for HCC progression [34].

To our knowledge, this is the first cohort study to evaluate the effects of glycemic burden, assessed by HbA1c level, on the recurrence and survival of early-stage HCC. We found that HCC patients with lower glycemic burden showed a lower recurrence rate and prolonged OS. The results also demonstrated that lower glycemic burden (HbA1c ≤ 6.5%) was an independent factor associated with lower recurrence and better OS. Additionally, analyses on glycemic burden subdivided into several categories suggested that there exists a dose–response relationship and poor glycemic control with HbA1c > 7.5% is an influential poor prognostic factor that may raise the recurrence risk as well as the mortality risk six times more.

We investigated the effect of glycemic burden rather than the presence of a history of DM on clinical outcomes of early-stage HCC in the present study. We used HbA1c levels instead of fasting blood sugar (FBS) levels to assess glycemic burden because HbA1c levels reflect glycemic control status more accurately than FBS levels. FBS level is a random glucose level at a point, and it fluctuates within a day, whereas HbA1c level is a more reliable indicator as it reflects an individual’s long-term glycemic control status during the preceding 2–3 months [35]. In fact, we set the cut-off point of HbA1c level as 6.5%, somewhat rigidly. First, one of the diagnosis criteria for DM is an HbA1c level higher than 6.5%. Secondly, most DM patients benefit from HbA1c levels below 7%, and a clinically significant difference is considered to be a change in HbA1c levels of 0.5% [36].

There are several limitations to this study. First, the subjects were patients with glycemic burden assessment. Actually, the current standard of care for HCC patients does not include serum HbA1c level assessment. Therefore, a large proportion of HCC patients were probably excluded from the beginning due to the lack of glycemic burden assessment because most patients took serum HbA1c level assessment as a part of DM management, not for the purpose of HCC management. Accordingly, there might have been selection or indication bias. Some papers have reported that antidiabetics may modify the incidence or prognosis of HCC in DM patients [37,38,39,40]. In this cohort, patients with HbA1c levels higher than 6.5% as well as patients with DM and HbA1c levels lower than 6.5% had received antidiabetic medication therapy or insulin therapy. However, we could not investigate the impact of antidiabetic medication on long-term outcome because the cohort size was relatively small to study the effects of different antidiabetic medications. Hence, what would be a potentially preferable antidiabetic medication for HCC patients remains an unsolved question. In the multivariable adjusted model, we tried to adjust potential confounders or mediators. However, other confounders such as alcohol and smoking history, the degree of liver fibrosis, or a family history of HCC were not considered. In addition, the serum HbA1c level used in this study is not a fixed variable as it can change during the follow-up and was not measured periodically because of the retrospective nature of this cohort study. Lastly, the studied cohort comprised of Asian patients. Generalization to other ethnicities is needed through validation. These limitations suggest that a well-designed prospective cohort study with a larger sample is required to see clinical relevance of measuring serum HbA1c levels in HCC patients for the purpose of risk management of HCC, not for the purpose of DM control. Nevertheless, until more robust data are available, the present study could give insight into the importance of assessing glycemic burden in HCC patients.

## 5. Conclusions

In conclusion, early-stage HCC patients with lower glycemic burden had a better prognosis than those with higher glycemic burden. These results demonstrate that glycemic burden can be a modifiable risk factor to improve outcomes of patients with early-stage HCC after curative treatment. Furthermore, a serum HbA1c target between 6.0% and 6.5% might be more preferential than a serum HbA1c target between 7.0% and 8.0%. Good glycemic control could be a therapeutic strategy for these patients to improve their clinical outcomes, which still requires further evaluation.

## Figures and Tables

**Table 1:** Comparison of baseline characteristics according to glycemic burden.

Variables	Low Glycemic Burden HbA1c ≤ 6.5% (N = 71)	High Glycemic Burden HbA1c > 6.5% (N = 66)	*p*-Value
Age, years	62.0 (55.0–66.0)	64.0 (56.7–69.2)	0.18
Male	58 (81.7)	56 (84.8)	0.65
Body mass index (kg/m^2^)	25.3 (22.6–27.9)	24.9 (23.4–26.3)	0.57
Hypertension	38 (53.5)	34 (51.5)	0.86
Dyslipidemia	35 (49.3)	42 (63.6)	0.12
Etiological liver disease			0.032
HBV	49 (69.0)	31 (47.0)	
HCV	3 (4.2)	5 (7.6)	
Others	19 (26.8)	30 (45.4)	
Child–Pugh score			0.46
5	48 (67.6)	33 (50.0)	
6–9	10 (14.1)	10 (15.2)	
Total bilirubin, mg/dL	0.7 (0.6–1.1)	0.6 (0.5–1.0)	0.23
Albumin, g/dL	4.5 (4.3–4.7)	4.3 (4.0–4.7)	0.19
AST, IU/L	30 (24–50)	34 (27–47)	0.22
ALT, IU/L	29 (20–41)	32 (21–50)	0.19
Prothrombin Time, INR	1.01 (0.97–1.06)	1.04 (0.97–1.11)	0.11
Platelet count (×10^3^/μL)	164 (123–206)	157 (108–219)	0.71
Tumor number			0.35
Solitary	67 (94.4)	59 (89.4)	
Multiple	4 (5.6)	7 (10.6)	
Tumor diameter, cm	3.0 ± 2.5	3.8 ± 3.0	0.08
Alpha fetoprotein, ng/mL	5.50 (2.30–17.7)	5.05 (2.95–35.6)	0.52
Treatment modality			0.34
Ablation	23 (32.4)	16 (24.2)	
Resection	48 (67.6)	50 (75.8)	

HbA1c = hemoglobin A1c; HBV = hepatitis B virus; HCV = hepatitis C virus; AST = aspartate aminotransferase; ALT = alanine aminotransferase; INR = international normalized ratio. Values are expressed as mean ± standard deviation, median with interquartile range, or frequency (percent).

**Table 2:** Risk of hepatocellular carcinoma recurrence according to glycemic burden.

Variables	Unadjusted HR (95%CI)	*p*-Value	* Adjusted HR (95%CI)	*p*-Value
HbA1c ≤ 6.5%	Reference		Reference	
HbA1c > 6.5%	2.05 (1.18–3.69)	0.013	2.66 (1.26–5.78)	0.011
HbA1c ≤ 6.0%	Reference		Reference	
HbA1c 6.0–7.5%	1.64 (0.79–3.65)	0.20	2.00 (0.78–5.55)	0.15
HbA1c > 7.5%	3.17 (1.53–7.03)	0.002	6.05 (2.31–17.5)	0.001

HR = hazard ratio; CI = confidence interval; HbA1c = hemoglobin A1c. * Hazard ratio was adjusted for age (per year), sex, tumor size (per cm), tumor number, Child–Pugh score (5 vs. 6–9), etiological liver disease (HBsAg positivity), and treatment method (resection vs. ablation).

**Table 3:** Risk of overall mortality according to glycemic burden.

Variables	Unadjusted HR (95%CI)	*p*-Value	* Adjusted HR (95%CI)	*p*-Value
HbA1c ≤ 6.5%	Reference		Reference	
HbA1c > 6.5%	1.61 (0.92–2.89)	0.10	2.33 (1.10–5.08)	0.029
HbA1c ≤ 6.0%	Reference		Reference	
HbA1c 6.0–7.5%	1.41 (0.67–3.14)	0.37	2.36 (0.89–6.86)	0.095
HbA1c > 7.5%	2.54 (1.22–5.64)	0.015	6.22 (2.36–18.1)	0.0004

HR = hazard ratio; CI = confidence interval; HbA1c = hemoglobin A1c. * Adjusted for age (per year), sex, tumor size (per cm), tumor number, Child–Pugh score (5 vs. 6–9), etiological liver disease (HBsAg positivity), and treatment method (resection vs. ablation).

## Data Availability

The data of the current study are available from the corresponding author upon reasonable request.

## Abbreviations

HCC	Hepatocellular carcinoma
RFA	Radiofrequency ablation
DM	Diabetes mellitus
NAFLD	Non-alcoholic fatty liver disease
HbA1c	Hemoglobin A1c
BCLC	Barcelona clinic liver cancer
HBV	Hepatitis B virus
HCV	Hepatitis C virus
AST	Aspartate aminotransferase
ALT	Alanine aminotransferase
AFP	Alpha fetoprotein
IQR	Interquartile range
aHR	Adjusted hazard ratio
CI	Confidence interval
IGF-1	Insulin-like growth factor-1
PI3K	Phosphoinositide 3-kinase
mTOR	Mammalian target of rapamycin
JNK	c-Jun N-terminal kinase
MAPK	Mitogen-activated protein kinase
MEK	Mitogen-activated protein kinase
ERK	Extracellular signal-regulated kinase
